# The eighth International Forum on Stem Cells: virtual meeting, October 20–21, 2022

**DOI:** 10.1097/BS9.0000000000000147

**Published:** 2022-12-27

**Authors:** Brian Eyden, Xiaochen Wang

**Affiliations:** a The Christie NHS Foundation Trust, Manchester M20 4BX, United Kingdom; b State Key Laboratory of Experimental Hematology, Institute of Hematology & Blood Diseases Hospital, Chinese Academy of Medical Sciences, Tianjin, China

Cancer accounts for significant levels of human morbidity and mortality. Lymphoma and leukemia are representative of the blood cancers. This dictates the moral imperative for fundamental research leading to clinical applications and therefore better outcomes, but also for conferences of transnational scope, such as the International Forum on Stem Cells, to share knowledge and experience.

The first International Forum on Stem Cells (IFSC) was held in Tianjin, China, in 2008. Subsequent biennial meetings alternately focused on stem cells in general and hematopoietic stem cells (HSC) more particularly. The inaugural and subsequent 6 meetings were held on-site in Tianjin, the home-city of the meeting sponsor, the Institute of Hematology & Blood Diseases Hospital, Chinese Academy of Medical Sciences (IHCAMS). Many internationally renowned stem-cell researchers have been speakers or conference co-chairs for these meetings. Typically, >500 scientists and students have come to Tianjin to contribute to and learn from each of these gatherings.^1^ Owing to the coronavirus pandemic, the 2020 conference had to take on a virtual format; about 1600 scientists, clinicians, and students, nevertheless, watched the meeting online.

The virtual IFSC2022 had an audience of >3500, and the online live stream had >30,000 hits. It was co-chaired by Professor Tao Cheng of IHCAMS (Tianjin, China) and Professor Margaret Goodell (Houston, USA). The program included 3 keynote speeches, as well as plenary sessions devoted to the following topics: lineage and differentiation, regulation, development, diseases, maintenance, technologies, the microenvironment, and therapy and translation.

IFSC2022 attracted a global participation, from China, the United States, the United Kingdom, Australia, Israel, Italy, Switzerland, and Japan.

The keynote speakers were **Professor Jim Palis** from the University of Rochester (USA); **Professor Alan D’Andrea** from the Dana-Farber Cancer Institute of Harvard University (USA); and **Professor Bertie Göttgens** from the University of Cambridge (the United Kingdom).

**Professor Palis** gave the opening keynote speech entitled “Making blood before a blood stem cell.” He described how robust HSC-independent waves of primitive and definitive erythroid progenitors emerged in the mammalian yolk sac, and how reactive oxygen species (ROS) and erythropoietin could activate *signal transducer and activator of transcription 3* specifically in primitive erythropoiesis. At embryonic day 10.5, primitive erythroblasts, which comprised about 50% of cells in the mouse embryo, were required for embryonic survival. Erythromyeloid progenitors emerged by endothelial-to-hematopoietic transition (EHT) and seeded the fetal liver to generate massive numbers of red blood cells (RBCs) to sustain mouse embryos to birth. BMI1 regulated in vitro self-renewal of murine and human erythroblasts, laying the groundwork for cultured RBCs to meet blood banking needs.

**Professor D’Andrea** delivered the second keynote speech, entitled “Bone marrow failure: Fanconi anemia and aplastic anemia.” The audience were reminded that Fanconi anemia is an extremely rare, autosomal recessive genetic disease characterized by developmental defects; bone marrow failure occurred by 5 years of age, and the children are susceptible to cancers such as leukemia and squamous-cell carcinoma. Partly, this is due to hypersensitivity to DNA crosslinking agents, and now 23 different complementation groups are known. The 23 FANC proteins co-operate in a common DNA crosslink repair pathway called the FA/BRCA pathway. The molecular pathogenesis of Fanconi anemia results from increased p53 expression, hyperactive TGFβ signaling, and increased Myc expression. In fact, new treatments for Fanconi anemia include gene therapy (GT) and the use of TGFβ inhibitors. Some of these results have implications outside hematopathology: in individuals other than those with Fanconi anemia, biomarkers of the FA/BRCA pathway can be used to predict the cisplatin or PARP1 (poly ADP ribose polymerase 1) inhibitor sensitivity of certain cancers.

The third and final keynote speech and the presentation that closed the meeting was given by **Professor Göttgens**. In “A new time and single cell resolved reference for hematopoiesis,” he introduced the new “kinetoscope model” of hematopoiesis, which provided single-cell resolution to observe unperturbed hematopoiesis. After reviewing the limitations of the classic hematopoietic tree model defined by flow cytometry and single-cell RNA-Seq data with its snapshot measurements lacking temporal information, he described the combination of persistent labeling (CreERT2, driven by the HSC-specific Hoxb5 gene) with time-series single-cell RNA-Seq to allow label-propagation analysis of the downstream progeny during steady-state hematopoiesis. Professor Göttgens mentioned that single-cell expression patterns could be coupled with dynamic changes in differentiation and growth speeds. By taking advantage of the available molecular information, new continuous models were also constructed to associate gene expression changes with cell behavior, thus directly connecting tissue and cellular behavior with the underpinning layer of molecular processes. By comparing datasets with single-cell transplantation data from Fang Dong’s group (from IHCAMS), transplanted stem-cell progeny were analyzed and significant upregulation of differentiation rates in neutrophils was pinpointed; in this way, the new model could quantify the impact of perturbations. Finally, Professor Göttgens looked forward to applying the quantitative tissue-scale approach to human disease. His work showed the hematopoiesis paradigm shift from traditional qualitative models with limited predictive capabilities to those with integrative and quantitative characteristics.

In the plenary sessions, 30 speakers brought new data and insights into a range of topics of current importance.

**Professor Emery Bresnick** (University of Wisconsin, USA) opened the session on *Lineage and Differentiation*, chaired by Jun Wei and Shuquan Rao, with a paper entitled “From human clinical genetics to leukemia predisposition-generating networks.” He discussed a patient with leukemia-predisposition syndrome (deficiency in the transcription factor, GATA-2) with a germline GATA-2 variant that inserts nine amino acids (AAs) between the two zinc-fingers (9 aa-Ins). Professor Bresnick revealed by genomic technologies how the insertion impacted GATA-2 function genome-wide. 9 aa-Ins was severely defective, with activation more impaired than repression. As for cellular signaling, hematopoietic-disrupting networks involving cytokine and pattern-recognition receptors were identified in GATA-2 deficiency. GATA-2 orchestrated diverse signaling processes that may inform GATA-2 deficiency syndrome phenotypes. It also endowed progenitors with the capacity to sense extrinsic stimuli and create feedback loops that controlled the genome, GATA-2 and cell-state transitions. The results established principles underlying GATA factor function, and comparable systems were being deployed to elucidate additional predisposition mechanisms.

**Professor Shalin Naik**, from The Walter and Eliza Hall Institute of Medical Research (Parkville, Australia), continued the session with a talk entitled “Towards revised (mathematical) models of hematopoiesis using cellular barcoding.” Professor Naik used cellular barcoding, a technique in which single cells can be labeled with unique nucleic acid sequences, termed *barcodes*, to track them spatially and temporally. By sending cell barcoding in microRNA format, a new lentivirus-based barcoding library was developed to investigate progenitor cell heterogeneity based on the revised “continuous” model of hematopoiesis. Recently, a lymphoid primed progenitor was identified by this technique. Moreover, it was not lineage plasticity but clonal tuning that accounted for the change in cell number during emergency hematopoiesis. By combining single-cell RNA sequencing and cell barcoding, Professor Naik’s team developed “SIS-seq,” which allowed the matching of genes related to lineage commitment with concrete cell fate. The technique can also be used to define the function of a given gene, instances being the *Bcor* and *Slpi* genes. Professor Naik’s team also studied the clonal contribution of defined hematopoietic stem and precursor cell (HSPC) subsets in vivo and in a native environment over time. Multistage lentiviral barcoding and transplant assays indicated that when going down the hierarchy, progenitor cells lost their clonal size, and their clonal equipotency shifted down. Other work consisted of the generative agent-based mathematical model in “fate space,” built to predict cell fate onto developmental time, as well as native hematopoiesis using the LoxCode mouse, which enriches the barcode diversity.

The presentation by **Professor Lihong Shi**, from the Chinese Academy of Medical Sciences, was entitled “Single-cell transcriptomic analysis identifies an immune-prone population in erythroid precursors during human ontogenesis.” One of the most interesting points was the classification of primitive erythroid cells into four functionally different subgroups. Primitive erythropoiesis first occurs in the yolk sac; definitive erythropoiesis then occurs in fetal liver; later, bone marrow becomes the main erythropoietic site. To investigate functional heterogeneity, Professor Shi’s group sorted erythroid precursors from fetal liver, umbilical cord blood, and adult bone marrow into 4 main clusters—C1, C2, C3, and C4. C1 genes were associated with mRNA catabolic processes, translational initiation and RNA splicing, and in yolk sac, they featured cholesterol biosynthesis and glycolysis. C2 cells expressed MKi67, CDK1 and CDC27, and in the fetal liver, they showed active cell division. C3 cells were associated with oxygen transport, with highly expressed hemoglobin genes, suggesting they were more mature than C1 or C2 cells. Most interestingly, and uniquely, C4 cells showed features of antigen processing and presentation in their expression of *SRGN* and *NFKBIA*. C1, C2, and C3 were regarded as classical erythroid cells, C4 as immune-prone erythroid precursors. Regulatory network analysis and CD63 expression in these clusters were also described.

The session on *Regulation*, chaired by Hideo Ema and Jia Yu, opened with a talk prepared by **Professor Linheng Li** (Stowers Institute for Medical Research, Kansas City, USA) entitled “Hematologic stem cells and niche interactions at the fetal stage.” It was first emphasized how essential HSCs and intestinal stem cells were for human health. An HSC niche was identified in 2003, but whether and how HSC subpopulations were regulated by bone marrow remains unclear. Professor Li’s work distinguished reserve HSCs (rHSCs) from primed HSCs (pHSCs) based on their response to chemotherapy, and examined how they were dichotomously regulated by bone marrow niches. rHSCs were preferentially maintained in the endosteal region enriched in N-cadherin-positive (N-cad^+^) cells in homeostasis and post chemotherapy.

Fetal liver HSC-niche representative gene expression was described. Using cutting-edge spatial transcriptomics, Professor Li’s team analyzed HSCs and niche cells in 14.5 day fetal liver; N-cad^+^ cells constituted a major niche in maintaining HSCs, while other potential niche cells were thought to be candidates for their expansion. The knocking-out of *Cxcl12* or *Scf* in N-cad^+^ cells led to the migration of HSCs toward other potential niche cells, such as macrophages and megakaryocytes. It was thought that this might induce HSC expansion and biased differentiation, suggesting that niche determined the lineage fate of HSCs.

The presentation by **Professor Guoji Guo** from Zhejiang University, entitled “Mapping cell landscapes at the single-cell level,” illustrates how advances in our understanding of human disease come not only from new data but by developing novel technologies. Professor Guo’s team used a single-cell approach to understand the genome, transcriptome, proteome, and epigenome of cells. They used microwell sequencing technology to study tens of thousands of individual cells, combined with mRNA barcoding, such that each cell had its own barcode and unique molecular identifier. Technical refinements enabled one million individual cells to be analyzed in one sequencing run. This produced a database for the computer to use artificial intelligence—a deep-learning-based strategy, Nvwa—and learn how a genome encodes cellular landscapes. From such analyses, 100 major clusters of the human cellular landscape were identified, which were refined into 843 subclusters. For example, clusters 5, 8, and 12 were immune-activated stromal cells which expressed IL family and CXCL family proteins. Using this database and landscape, changes across species and during whole lifespans could be studied. In one instance, 2500 cellular states in a mammal across its lifespan were identified—not only “classical” cell types like immune, epithelial, and endothelial cells (ECs) but also transitional cell types, such as epithelial immune cells, endothelial immune cells, and mesenchymal immune cells. Additional work discussed included how cell types were regulated across species (here the transcription factor, Xbp1, was important), and the role of artificial intelligence in interpreting the trajectory from genome to cellular landscape, where Nvwa appeared to be learning the mechanism of how noncoding sequences affected gene activation and repression across lineages and species.

The session was continued by **Professor Junke Zheng**, from the Shanghai Jiao Tong University. His talk, “Metabolic regulation in hematopoieis and leukemogenesis,” began with work on an NADH/NAD^+^ sensor (“SoNar”) to indicate NADH/NAD^+^ levels in living leukemic cells. SoNar-high cells were more glycolytic, enriched for higher leukemia-initiating cell (LIC) frequency, and developed leukemia much more quickly than SoNar-low counterparts in an MLL-AF9-induced murine acute myeloid leukemia (AML) model. SoNar-high cells mainly homed to and located in the hypoxic endosteal niche. In addition, B-acute lymphoblastic leukemia cells (B-ALL cells) showed a preference for oxidative phosphorylation as the main energy source and localization in the bone marrow vascular niche. B-ALL-LICs were more enriched in SoNar-low cells, which were more resistant to Ara-C treatment. CREB-mediated PDHX and CDA pathways fine-tuned the metabolic properties and drug resistance of B-ALL cells. iNap-high AML cells had enhanced colony-forming abilities and were enriched in LICs. These findings provided important clues for understanding the mechanisms for regulating cancer-cell fates and for a potential metabolic target for leukemia treatments.

**Dr Kuangyu Yen**, from the Chinese Academy of Medical Sciences, rounded off the session with a presentation entitled “Transcriptome dynamics reveal stepwise tipping points.” She focused on EHT, exploring the dynamics and regulation of tipping points, which were peaks in potential between two stable states. Combining single-cell transcriptomics, in silico simulations, and functional validation, the precursor of the hemogenic endothelial (pre-HE) stage and the type I pre-HSC (T1 pre-HSC) stage represented 2 tipping points in EHT. The first tipping point may be overcome via upregulating the master regulators, Cebpb and Spi1, to facilitate the transition through the second tipping point. Cebpb and Spi1 were the main regulators that governed T1 pre-HSC. Knockdown of Cebpb reduced HSPC formation and reverted hematopoietic cells to HE-like cells, demonstrating that manipulating the tipping point can reverse cell fate. The 2 tipping points in EHT may reflect 2 steps in cell-fate commitment—specification and determination/maturation. These findings shed new light on the dynamics and drivers of cell fate transition.

The session on *Development*, chaired by Bing Liu and Weijun Pan, was opened by **Professor Yu Lan** of Jinan University, China, who discussed “Heterogeneity and diversification of early embryonic endothelial cells.” Professor Lan emphasized that during early embryonic angiogenesis, the process of endothelial diversification and the molecular events underlying arteriovenous fate settling remains largely unresolved in mammals. Using single-cell transcriptomics, the landscape of ECs was constructed during the time of key vasculogenic and angiogenic events in both mouse and human embryos. First, HSC-competent hemogenic ECs were precisely captured by the newly constructed Neurl3-EGFP reporter mouse model, with further enrichment by a combination of surface markers (Procr^+^Kit^+^CD44^+^, PK44). Surprisingly, the endothelial-hematopoietic dual potential was rarely but reliably witnessed in the cultures of single hemogenic ECs. Two distinct arterial EC types were identified—the major artery ECs and the arterial plexus ECs—in addition to the unexpectedly divergent arteriovenous characteristics among ECs that are located in the vascular plexus intra-embryonically. Using computational prediction and further lineage tracing of venous-featured ECs with a newly developed Nr2f2CrexER mouse model and a dual recombinase-mediated intersectional genetic approach, early and widespread arterialization from the capillaries with notable venous characteristics was revealed. The findings provided unprecedented and comprehensive details of endothelial heterogeneity and lineage relationships in the early stages of angiogenesis, and established a new model for the arteriogenesis behavior of the early intra-embryonic vasculature.

**Professor Tomomasa Yokomizo**, from the Tokyo Women’s Medical University, then discussed “Independent origins of fetal liver hematopoietic stem and progenitor cells.” He raised the intriguing question: how do HSCs accomplish self-renewal and differentiation?—2 seemingly opposing tasks—within a mere 4-day window from 10.5 to 14.5 days of embryonic life? Professor Yokomizo used tamoxifen-induced *Hlf^creERT2^ROSA^tdTomato^* mice in a time-course kinetic analysis in vivo to reveal a new hematopoietic hierarchy. He found that fetal HSCs contributed minimally to progenitors and mature cells. The tracing results indicated that HSCs and progenitors were generated independently from pre-hematopoietic stem and progenitor cells (pre-HSPCs). The parallel emergence of HSCs as well as various progenitors from HLF^+^KIT^+^ precursors implied a heterogeneity within the pre-HSPC population. By comparing different expression genes and taking into account the results from other experiments, it was shown that intra-embryonic EVI1^hi^ cells preferentially generated HSCs while EVI1^−^ cells preferentially generated progenitors, suggesting that the gradient of EVI1^hi^ expression regulates the fate of pre-HSPCs.

**Professor Jiaxi Zhou**, from the Chinese Academy of Medical Sciences, presented a paper entitled “Megakaryocyte single-cell transcriptomics and beyond,” in which he explored the molecular and functional heterogeneity of megakaryocytes (MKs) throughout mammalian life, for example, in relation to their formation from HPSCs. Dr. Zhou’s team had already revealed a comprehensive single-cell transcriptomic landscape of human MKs, finding a cellular heterogeneity with distinct metabolic and cell-cycle signatures. They identified a novel subpopulation of immune-surveillance MKs, marked by CD148 and CD48 in vivo and in vitro and able to generate special platelets. More recently, they provided the first spatio-temporal single-cell transcriptomic landscape of mammalian MKs, in studies on yolk sac, liver and bone marrow. These findings may offer the promise of advances in a large-scale platelet production and transfusion therapy in precision medicine.

The presentation by **Professor Elaine Dzierzak** from the University of Edinburgh, Scotland, United Kingdom, on “The endothelial cell transition to hematopoietic stem cell, progenitor cell or other?” focused on the three de novo generations of hematopoietic cells in the embryo—primitive cells; definitive progenitor cells; and definitive HSCs. Within a short period, HSCs originated from aortic cells in the process of EHT. Transcription factors such as Gata2 and Runx1 regulated the process via extrinsic signals such as Notch, TGF, and BMP from the surrounding developmental niche. EHT also generated definitive HPCs. Similar regulators participated at both stages, raising the question: What mechanistically determined HPC or HSC during EHT? Vital confocal time-lapse imaging revealed highly dynamic Gata2 expression changes in cells during EHT. Moreover, Gata2 pulsatile expression behavior was dramatically altered in Gata2-haplo-insufficient embryonic aortic cells, which underwent fewer EHTs and had less hematopoietic cell potential. This suggested that Gata2 was the rate-limiting intrinsic regulator influencing the transition to an HSC or an HPC fate. Studies on the aortic niche revealed that rapid transient cell–cell interactions between yolk sac HPC-derived macrophages and aortic ECs take place in transition to HPCs and HSCs. Localization and molecular and functional analyses of extrinsic factors from macrophages identified a new pro-inflammatory macrophage subset unique to the embryonic stages of hematopoietic development. These results improved our understanding of the dynamics of regulators specific for the generation of HSCs and HPCs in the embryo, and provided insights into how these mechanisms can be harnessed to generate HSCs ex vivo, which, in turn, may have therapeutic potential.

The session on *Diseases*, chaired by Hudan Liu and Jun Shi, was opened with a presentation by **Professor Jianjun Chen**, from City of Hope, California, USA, entitled “METTL16 drives leukemogenesis and leukemia stem-cell self-renewal by reprogramming BCAA metabolism.” The talk introduced the N6 methyladenosine (m6A) modification machinery, m6A being the most prevalent internal modification in mRNA, regulating mRNA fate and gene expression post-transcriptionally. Dysregulation of m6A leads to various types of cancer, including AML. m6A methylation is mainly installed by the METTL3-METTL14 complex, and both METTL3 and METTL14 are linked to AML initiation and progression. However, double depletion of METTL3 and METTL14 resulted in only a 50% to 60% decrease in m6A, and over 50% of the m6A residues did not coincide with the binding sites of METTL3 and METTL14 at the whole transcriptome level; this hinted at the existence of additional m6A writer proteins. Professor Chen’s team also analyzed 2 genome-wide CRISPR-Cas9 screening data sets and their in-house-specific CRISPR-Cas9 screening data; METTL16 was found to have a robust oncogenic role in AML development/maintenance and leukemia stem cell (LSC)/LIC self-renewal, as demonstrated in bone marrow transplantation models, human-in-mouse xenograft models and patient-derived xenograft models. Other results involved branch-chain AA (BCAA) transaminase 1 (BCAT1) and BCAT2 expression in m6A, highlighting the METTL16/m6A/BCAT1-2/BCAA axis in leukemogenesis and the essential role of METTL16-mediated m6A epitranscriptome and BCAA-metabolism reprogramming in leukemogenesis and LSC/LIC maintenance.

**Professor Haojian Zhang**, from Wuhan University, China, gave a talk on “The molecular mechanisms of leukemia stem cell maintenance.” In addressing how genetic and epigenetic alterations occur, and how LSCs maintain their function, next-generation sequencing data showed that many genetic alterations occurred in epigenetic-related genes such as TET1, TET2, IDH2 and DMT3; this suggested that chromatin stage controlled AML development. ATAC-seq identified pre-LSCs, LSCs and blasts. Compared with normal hematopoietic stem progenitor populations, significant chromatin remodeling during leukemogenesis was observed, featuring 2 clusters—Cluster1, showing gain of accessibility, and Cluster2 showing loss. Cluster1 genes included that for ALKBH5, which was important in m6A (discussed already by Professor Jianjun Chen). In knockout mice, deletion of ALKBH5 significantly delayed AML development and was also required for maintaining functional LSCs in AML. Professor Zhang further reported findings on the ALKBH5-AXL single pathway as a potential strategy for eradicating LSCs; on the multi-functional protein, YBX1 is essential for the survival of human and mouse myeloid leukemia cells; on a new technology called *SLIM-seq*, which enabled mRNA profiling on extremely limited numbers of cells; and on the BMI1 gene, as a functional downstream target of IGF2BP2 in HSCs.

**Professor Liran Shlush**, from the Weizmann Institute of Science, Israel, opened his presentation entitled: “Mutation mechanisms in clonal hematopoiesis” by explaining that human aged HSPCs undergo clonal expansion through acquiring somatic mutations, the phenomenon known as *age-related clonal hematopoiesis* (ARCH; *clonal hematopoiesis* being abbreviated here as CH). While the somatic pre-leukemic mutations which characterize ARCH are mostly non-synonymous single-nucleotide variants, insertions and deletions are less common, and their mutational mechanisms are less well understood. The genomic regions around current deletions in myeloid malignancies were analyzed, and micro-homology-based (MH-based) signatures in CALR, ASXL1 and SRSF2 loci were identified. These deletions resulted from double-strand-break repair by a PARP1-mediated, micro-homology-mediated end-joining pathway (MMEJ). Importantly, evidence was provided that recurrent MH-based deletions originated in pre-leukemic HSCs suggesting an MMEJ predominance in CH. While polymerase theta was considered a key component in MMEJ repair, it also seemed to be the case that pre-leukemic MMEJ (preL-MMEJ) deletions could be generated in POLQ knockout cells. However, inhibition of the replicative polymerases (which are co-expressed with PARP1 in human HSCs) led to a significant reduction in preL-MMEJ. In summary, the data indicated an association between polymerase-theta-independent MMEJ and CH, and shed light on the mutational mechanisms of the earliest steps in leukemia evolution.

Another group focusing on CH was that of **Professor George Vassiliou** from the University of Cambridge, United Kingdom, who presented a paper entitled “The natural history of clonal hematopoiesis.” Instead of maintenance, Vassiliou’s group focused on CH associated with age, with its increased risk of myeloid leukemia and some non-hematological diseases. They developed a predictive model to identify a significant proportion of individuals that progressed to AML. They tracked 395 people with a median age of 69 over an 8- to 15-year period, and in-depth sequenced blood DNA samples with a myeloid panel of 53 genes. Strikingly, they found that in some age groups, TET2 mutations were more common than DNMT3A, the most studied of CH mutations. They also found different lifelong behaviors of different types of CH: for example, DNMT3A clones grew early, then decelerated; TET2 clones stayed stable and splicing gene clones started later in life and grew quickly, which explains the different phenomena of the changing prevalence of different genes.

Opening the session on *Maintenance*, chaired by Fang Dong and Fang Ni, **Professor Margaret Goodell**, from the Baylor College of Medicine, shared her laboratory’s latest research in a talk entitled: “Mechanisms driving clonal hematopoiesis.” She described three important genes: PPM1D, SRCAP, and DNMT3A. PPM1D, also called WIP1, functioned as a negative regulator in the response to DNA damage, and was classified as a moderately strong driver in CH. Professor Goodell’s team showed that PPM1D mutations were highly enriched in patients exposed to certain chemotherapy regimens. Although it seemed to work through suppression of p53-DDR, it was not identical to p53 mutations. The second gene described was SRCAP, the core component of the nucleosome remodeling complex. It was found that the competitive advantage of SRCAP-m cells was largely observed even in the absence of drug treatment in vivo. It was proved that the roles of SRCAP in CH were more than promoting DNA repair to have this competitive advantage, but also upregulating chromatin remodeling genes. Last, a brief description was given of DNA methyltransferase 3A (DNMT3A), the best known driver of CH. HSCs in the DNMT3A-m cells could obtain bias self-renewal ability, and different mutations in DNMT3A gave rise to different levels of DNMT3A activity. In summary, each of the 3 different genes had a distinct strategy in CH.

DNMT3A was also the subject of presentations by **Professor Pengxu Qian**, from Zhejiang University, China, and by **Professor Tianpeng Gu**, from the Chinese Academy of Medical Sciences. In Professor Qian’s talk—“Development of a novel therapeutic approach against DNMT3A-mutant acute myeloid leukemia”—he reminded the audience how DNMT3A was the most frequently mutated gene in ARCH, and was associated with increased risk of hematologic malignancies including AML. Although DNA-damaging chemotherapy agents have improved outcomes for DNMT3A-mutant AML patients, the overall survival and the recurrence-free survival remain suboptimal, and no targeted therapy is currently available; this highlights the need for further study of how DNMT3A mutations affect the AML phenotype. It was found that cell-adhesion-related genes were predominantly enriched in DNMT3A-mutated cells. By screening a series of carbon nanomaterials, it was found that the two-dimensional graphdiyne oxide (GDYO) exhibited an anti-leukemia effect specifically against the DNMT3A-mutant AML cells. GDYO had better dispersion potential in physiological solution than graphene oxide owing to its higher ionization status, resulting in improved bioactivity. Mechanistically, GDYO directly interacted with β2 (ITGB2) and c-type mannose receptor (MRC2), which are highly expressed in DNMT3A-mutant AML cells, and participated in cellular adhesion, facilitating the attachment and cellular uptake of GDYO. Furthermore, GDYO bound to actin and prevented actin polymerization, thus disrupting the cytoskeleton and eventually leading to cell differentiation and apoptosis. Finally, the in vivo safety and therapeutic potential of GDYO against DNMT3A-mutant AML cells was demonstrated. Collectively, these findings showed that GDYO was an efficient and specific drug candidate against DNMT3A-mutant AML cells.

In the talk given by **Professor Tianpeng Gu**—“The N-terminal domain of DNMT3A recognizes H2AK119ub and is required for post-natal development”—DNA methylation was emphasized as a heritable epigenetic modification deposited on cytosine in CpG dinucleotides in the mammalian genome by DNA methyltransferases (DNMTs). Knockout mouse models showed that DNMT3A was essential for post-natal development and was recognized in multiple nervous system processes. DNMT3A mutations have been frequently identified in hematologic malignancies, and, more recently, in human developmental disorders such as Tatton-Brown-Rahman syndrome. Two isoforms of DNMT3A, which differed in the presence of a 219 AA N-terminus in the full-length DNMT3A, were differentially expressed from stem cells to somatic tissues; however, their individual functions remain largely uncharacterized. The long isoform, DNMT3A1, but not the short DNMT3A2, was found to be essential for mouse post-natal development. DNMT3A1 bound to and regulated bivalent neurodevelopmental genes in the brain, and Dnmt3a1 knockout perinatal lethality could be partially rescued by DNMT3A1 restoration in the nervous system. It was further shown that the intrinsically disordered N-terminus of DNMT3A1 was required for normal development and DNA methylation in DNMT3A1-enriched regions. Mechanistically, a putative alpha-helix motif embedded in the N-terminus bound to histone H2AK119 mono-ubiquitination (H2AK119ub), in all likelihood mediating the recruitment of DNMT3A1 to Polycomb-regulated regions. These data demonstrated an isoform-specific role for DNMT3A1 in mouse post-natal development and revealed the N-terminus as a necessary regulatory domain for DNMT3A1 chromatin occupancy and functions in the nervous system.

The presentation by **Professor Dachuan Zhang**, of Shanghai Jiao Tong University, China, on “The microbiota-iron axis regulates hematopoietic stem cell fate decisions under stress” emphasized how so many components of the human body, traditionally regarded as separate and independent, are in fact functionally co-ordinated. Professor Zhang started his talk by underlining the idea that host microbiota crosstalk was essential for the production and functional modulation of blood cell lineages. Whether, and if so how, the microbiota influences HSCs remained unclear, however. The microbiota was shown to regulate the self-renewal and differentiation of HSCs under stress conditions by modulating local iron availability in the bone marrow. In microbiota-depleted mice, HSC renewal was enhanced during regeneration, while the commitment towards differentiation was dramatically compromised. Mechanistically, microbiota depletion selectively impaired the recycling of RBCs by bone marrow macrophages, resulting in reduced local iron levels without affecting systemic iron homeostasis. Limiting iron availability in food (in vivo) or in culture (ex vivo) or by CD169^+^ macrophage depletion-enhanced HSC self-renewal and expansion. These results revealed an intricate interplay between the microbiota, macrophages and iron, and their essential roles in regulating critical HSC fate decisions under stress.

The session *Technologies*, chaired by Hong Wang and Ping Zhu, underlined how, in the last decade, single-cell technologies as well as multi-omics technologies have made significant progress, uncovering the heterogeneity of previously defined HSCs. From the embryonic through to the adult stage to aging, single-cell studies have enabled the tracing of stem cell origins, demonstrating different cell differentiation routes during development, as well as identifying novel cell populations. For both benign and malignant diseases, single-cell omics technology has begun to reveal cellular complexity, map clonal evolution, and dissect microenvironmental systems, thereby greatly broadening the understanding of both hematopoiesis and disease development. Moreover, by single-cell technologies, there have also been advances in the molecular mechanisms for relapse and therapeutic targets of various diseases.

The session began with a talk by **Professor Rong Fan**, from Yale University, USA, entitled: “Spatial multi-omics driving the next wave of biomedical research.” Professor Fan focused on the study of multicellular systems which need to be conducted in the native tissue context defined by spatially resolved molecular profiles. His research was aimed at better understanding the role of spatial heterogeneity in biological, physiological, and pathological processes. He discussed the emergence of a whole new field—“spatial omics”—and then focused on a new technology platform called *Deterministic Barcoding in Tissue* (DBiT) for spatial-omics sequencing developed in his laboratory in recent years. Professor Fan’s department has co-mapped the whole transcriptome and proteome (~300 proteins) pixel-by-pixel directly on a fixed-tissue slide in a way compatible with clinical tissue specimens including the formalin-fixed paraffin-embedded material. It has been applied to the study of developing mouse brain, human brain, and human lymphoid tissues associated with normal physiology, diseases, and aging. Professor Fan’s research recently opened up another new field—*spatial epigenomics*—by developing multiple DBiT-based spatial sequencing technologies for mapping chromatin accessibility (spatial-ATAC-seq), histone modification (spatial-CUT&Tag), or further combined with transcriptome or proteins for spatial co-profiling. These new technologies allowed the visualization of gene expression regulation mechanisms pixel by pixel directly in mammalian tissues with a near single-cell resolution.

**Professor Weike Pei**, from Westlake University, China, gave a presentation: “Genetic lineage tracing to explore the blood and immune system in health and disease.” He opened his talk by remarking on the need to understand how cells make decisions in their differentiation into specialized cells. To address this question, it was crucial to resolve developmental histories and cell fates, also known as *lineage tracing*. Previous approaches were limited either by invasive manipulation, that could alter cell physiology, or by low resolution, which was unable to dissect the complexity of the organism. Therefore, a Cre-driven endogenous DNA barcoding system—Polylox—was developed to achieve high-resolution, noninvasive lineage tracing in mice in vivo. Professor Pei’s team traced how various types of blood and immune cells were generated from HSCs in situ, and discovered that HSCs consisted of subsets with different fate patterns, which were designated as multi-lineage, myeloid-restricted and differentiation-inactive. A step toward understanding cell-fate decisions required the dissection of molecular programs driving these divergent cell fates. The Cre-driven RNA barcoding system generated expressed DNA barcodes in situ, enabling paired analysis of cell fates and transcriptomes at the single-cell level. From this barcoding system, HSC clones were transcriptionally more similar in the same fate than other HSC clones. Fate-associated gene signatures were characterized, providing potential determinants for HSC fate choice. These studies offered unique entry points for building lineage relationships and defining cell-fate regulators in an unbiased and systematic manner, with far-reaching implications beyond the blood and immune system.

**Professor Aibin He**, from Peking University, also working with single-cell technology, presented a discussion on “Genome-coverage single-cell histone modifications in mouse pre-implantation development.” He started by highlighting how epigenetic resetting was important in mammalian pre-implantation development. However, the crosstalk during assembling the diverse histone modifications in this process at the single-cell level remained elusive. Professor He introduced TACIT, a single-cell high-sensitivity assay for measuring protein-DNA interactions with genome-coverage. TACIT, producing ultra-high reads per cell, was applied to individual pre-implantation cells at zygote, 2-, 4-, and 8-cell embryo, morula and blastocyst stages to profile a panel of histone modifications—H3K4me1, H3K4me3, H3K27ac, and H3K36me3 as active marks; H3K9me3 and H3K27me3 as repressive marks and a histone variant H2A.Z. H3K27ac was found to be a “pioneer” mark priming for ZGA at the 2-cell stage. By integrating these histone marks into a synthetic single cell, it was shown that the acquisition of repressive chromatin states was linked to the exit of totipotency. Importantly, the identification of key cell-fate transition and regulation from multi-modality analysis is otherwise impossible by using any single modality of histone codes. Collectively, this work presented a comprehensive single-cell landscape of epigenetic reprogramming during early mouse embryo development.

**Professor Timm Schroeder**, of the Swiss Federal Institute of Technology, ended the *Technologies* session, with a talk: “Long-term single-cell quantification: new tools for old questions.” He focused on the identification of a blood stem-cell fate regulator using single-cell tracking and omics, and cell-fate control by signaling dynamics. He pointed out that the major problems in finding cell-fate regulator molecules were “wrong populations” (cells analyzed long before or after the decision-making moments) and “impurity/no synchronization” within populations. Professor Schroeder’s team integrated single-cell omics with tracking and dynamics quantification to compare HSC daughter cell fate regulator detection. They employed and improved trackSeq for better control of confounders, and revealed that transcriptome divergence at HSC activation after asymmetric division included differential cell-cycle and adhesion regulation, which could be validated by functional experiments. As regards cell-fate control, Professor Schroeder’s team studied NFκB signaling. They conducted time-lapse imaging and tracking of single murine HSPCs from green fluorescent protein-p65/H2BmCherry reporter mice to quantify NfκB activity dynamics in response to TNFα and interleukin 1β. For the first time, they described cell-type characteristic NfκB dynamics in a primary mammalian tissue (blood) stem cell differentiation system. Time-lapse imaging, single-cell RNA sequencing, plus signaling manipulation showed that NfκB dynamics could influence cell behavior.

The session on *Microenvironment*, chaired by Hui Cheng and Bo Zhou, began with a presentation by **Professor Daniel Lucas**, from the Cincinnati Children’s Medical Centre, USA, entitled: “The anatomy of normal and stress hematopoiesis.” His team has developed new strategies to image the bone marrow and to generate a map of the anatomy of hematopoiesis at the single-cell level. They found that the basic anatomy of hematopoiesis was characterized by migration, multipotent and oligopotent progenitors migrating long distances from parent and sister cells, lineage-committed progenitors localizing to arterioles (for lymphoid progenitors) or sinusoids (myeloid and erythroid), whereas immature cells were generated in micro-anatomical structures with unique spatial and clonal architecture. Even under stress, hematopoiesis used the same micro-anatomical structures as the underlying steady state. On removal of the signal, the production lines reverted to homeostasis.

The session continued with a talk by **Professor Meng Zhao**, from the Sun Yat-sen University, China, with the title: “The metabolism and translation regulation of hematopoietic stem cells.” Professor Zhao reminded the audience that it was still incompletely understood how HSCs adapted their metabolism to maintenance and proliferation. His team demonstrated that homeostatic HSCs exhibited high AA catabolism to reduce cellular AA levels, which activated the GCN2-elF2a axis, a protein-synthesis-inhibitory checkpoint to restrain protein synthesis for maintenance. Furthermore, upon certain proliferation conditions, HSCs enhanced mitochondrial oxidative phosphorylation (OXPHOS) for higher energy production, but decreased AA catabolism to accumulate cellular AAs, which inactivated the GCN2-elF2a axis to increase protein synthesis and coupled with proteotoxic stress. Importantly, GCN2 deletion impaired HSC function in repopulation and regeneration. Mechanistically, GCN2 maintained proteostasis and inhibited Src-mediated AKT activation to repress mitochondrial OXPHOS in HSCs. Moreover, the glycolytic metabolite NAD^+^ precursor nicotinamide riboside accelerated AA catabolism to activate GCN2 and sustain the long-term function of HSCs. Overall, the study uncovered direct links between metabolic alterations and translation control in HSCs during homeostasis and proliferation.

In a talk entitled “Healthy and malignant hematopoiesis in the bone marrow: dynamic cells in an evolving environment,” **Professor Cristina Lo Celso** from Imperial College, London, United Kingdom, used single-cell microscopy tracking to visualize cells based on their expression of fluorescent reporters within the bone marrow and revealed the cellular dynamics driving hematopoietic regeneration. It has been reported that AML leads to loss of stroma and especially of endosteal HSC niches, and that acute *Plasmodium* infection leads to leaky bone marrow vasculature and acute loss of osteoblasts. Using intravital microscopy in a murine model of AML, Professor Lo Celso and her team showed that as malignant cells grow in the bone marrow, clusters of healthy cells enter the circulation through the vasculature which becomes leaky at earlier stages than previously thought. Her team also explored the role of extracellular matrix remodeling in regulating AML growth and differentiation, and the highly dynamic interactions between AML cells and immune cells. Multiple matrix metalloproteinases (MMPs), expressed by AML cells and the bone marrow microenvironment, can remodel extracellular matrix. Treatment with the MMP inhibitor prinomastat lessened cell ousting and vascular leakiness and affected AML cell migration and growth. Thus, the bone marrow microenvironment can be a therapeutic target to preserve and support HSCs and hinder leukemia growth.

**Professor Simón Méndez-Ferrer**, from the University of Cambridge, United Kingdom, rounded up the *Microenvironment* session, with a presentation entitled: Understanding and targeting the extrinsic HSC regulation in the myeloproliferative neoplasms.” His team emphasized the significant neuroendocrine influence on bone marrow, in which, for example, the brain regulates a peripheral stem-cell niche where mesenchymal stem cells (MSCs) play a key role in the normal HSC niche. Aging was associated with increased risk of myeloproliferative neoplasms (MPNs) and AML. Remodeling of bone marrow niches promoted myeloid-cell expansion during aging. Furthermore, AML cells co-opted energy sources and antioxidant defense mechanisms from HSC-niche-forming MSCs to survive chemotherapy. This suggests a potential importance of adjuvant niche-targeting therapies. MSCs integrated signals derived from the brain and the peripheral nervous system to regulate HSCs in a co-ordinated manner to meet organismal demands. Also, damage to this regulatory network was required for MPN development and could be a therapeutic target in myelofibrosis. The neuroendocrine regulation of bone marrow stem cells by noradrenergic signals or by sex hormones could be harnessed to offer novel therapeutic approaches for MPN. The latter has recently been tested in a Phase II, multi-center, single-arm clinical trial assessing tamoxifen’s safety and activity in reducing molecular markers of disease burden in MPN. The results suggest that tamoxifen can modulate the unfolded protein response and inhibit mitochondrial respiration and pathogenic JAK-STAT signaling in a subset of potentially prospectively identifiable patients.

The final session, *Therapy/Translation*, chaired by Qianfei Wang and Weiping Yuan, opened with a talk entitled “Enhanced anti-tumor activity of human iPSC-derived immune cells,” by **Professor Dan Kaufman**, of the University of California at San Diego, USA. His team used human induced pluripotent stem cells (iPSCs) to generate natural killer (NK) cells and macrophages and improve their anti-tumor activity. They derived iPSC-NK cells with novel NK-cell-specific chimeric antigen receptors (CARs), stabilized the expression of the FC receptor, CD16, and the deletion of intracellular CISH expression—all of which led to improved cell killing in hematologic malignancies and solid tumors. iPSC-NK cells were derived with deletion of the TGFbII receptor as a new approach to prevent tumor-cell-mediated inhibition of NK-cell killing. iPSC-derived macrophages were also engineered which expressed macrophage-specific CARs that improved killing of tumor cells. The combination of iPSC-derived NK cells and macrophages improved anti-tumor activity. These approaches offered new strategies to produce targeted “off-the-shelf” therapies to better treat and cure refractory malignancies.

The session continued with a talk by **Professor Luigi Naldini**, from the San Raffaele University, Italy, on “Advanced genetic engineering of hematopoiesis to treat human disease.” He emphasized how HSC GT by lentiviral vectors was providing benefits to patients with primary immunodeficiencies, hemoglobinopathies and storage disorders. Long-term follow-up has shown stable hematopoietic reconstitution by large numbers of corrected HSCs without signs of clonal expansion or exhaustion, leading to long-term clinical benefit. Gene editing may improve the safety of HSC GT by achieving in situ gene correction or targeted transgene integration. Professor Naldini’s team has reported the first targeted gene editing of human HSC followed by studies highlighting barriers to its efficiency, and novel strategies overcoming them. Homology-driven repair, however, remains limiting. The team reported that the choice of template can increase the efficiency and safety of the procedure. Furthermore, the emergence of base and prime editors that minimize or bypass the requirements for DNA double-strand break allowed editing single or few mutant nucleotides with limited activation of the DNA damage response. A further topic elaborated on was the making of space for the infusing cells without relying on genotoxic conditioning. HSC mobilization enabled engraftment of donor cells which outcompete those in the circulation.

The final plenary session of IFSC2022 concluded with a presentation by **Professor Anskar YH Leung**, of the University of Hong Kong, China, entitled “Complex interplay between immune microenvironment and malignant hematopoietic cells – the TP53 mutated MDS/AML paradigm.” The TP53 mutation of HSCs and progenitor cells was underscored as being associated with myelodysplastic syndrome and AML (MDS/AML). Clinical and laboratory data were presented on the effects of the hypomethylating agent, decitabine, and the polo-like kinase 4 (PLK4) inhibitor on TP53-mutated MDS/AML. Both agents induced cell-intrinsic effects on leukemia cells and non-cell intrinsic effects on immune compartments. Combination treatment with PLK4 inhibitor and anti-CD47 antibody, which blocks the “don’t eat me” signal, enhanced anti-leukemia effects in vivo. The data emphasized the complex interplay between leukemia and the immune compartment on treatment with anti-leukemia agents in TP53-mutated MDS/AML and offered possibilities for future therapeutic strategies.

## CONCLUDING REMARKS

Although the IFSC2022 meeting had a virtual format, this had no negative impact on scientific communication, since, as Professor Tao Cheng commented, “Communication in the field of stem cell science does not stop.”

Furthermore, as quoted by Professor Orkin (mentioned by Professor Lappin in his IFSC2020 Meeting Report^2^):

“The only real voyage of discovery consists not in seeking new landscapes, but in having new eyes” (Marcel Proust).

Another way of expressing this “seeing with new eyes” is *using new technologies*, and several presentations in the IFSC2022 underlined how scientific imagination continues to power the emergence of novel techniques—single-cell transcriptomics; microwell sequencing technology combined with mRNA barcoding; the deep-learning-based strategy, Nvwa; mRNA profiling on extremely limited numbers of cells; the use of carbon nanomaterials such as graphdiyne oxide; and spatial epigenomics, for example. These will surely advance future research.

Any meeting is only a stepping stone in the continuing advancement of knowledge and ideas, and IFSC2022 played its part in providing a platform for researchers and clinicians to share ideas and data with the ultimate objective of developing new clinical applications. The sheer quantity of data and new ideas, however, can be overwhelming, the management of which demands the scientific insight to distill these data and ideas into those fewer paths most likely to profitably advance effective therapy; meetings such as the IFSC2022 will favor this process.

## ACKNOWLEDGMENTS

We would like to thank Professor Hideo Ema and Professor Guoguang Zheng for their comments, and Tao Cheng’s and Hideo Ema’s laboratory members for their meeting notes.
